# Mycotoxin Occurrence, Toxicity, and Detoxifying Agents in Pig Production with an Emphasis on Deoxynivalenol

**DOI:** 10.3390/toxins13020171

**Published:** 2021-02-23

**Authors:** Debora Muratori Holanda, Sung Woo Kim

**Affiliations:** Department of Animal Science, North Carolina State University, Raleigh, NC 27695, USA; dmurato@ncsu.edu

**Keywords:** aflatoxin, deoxynivalenol, mycotoxin detoxification, fumonisin, mycotoxin frequency, mycotoxin toxicity, pig

## Abstract

This review aimed to investigate the occurrence of mycotoxins, their toxic effects, and the detoxifying agents discussed in scientific publications that are related to pig production. Mycotoxins that are of major interest are aflatoxins and *Fusarium* toxins, such as deoxynivalenol and fumonisins, because of their elevated frequency at a global scale and high occurrence in corn, which is the main feedstuff in pig diets. The toxic effects of aflatoxins, deoxynivalenol, and fumonisins include immune modulation, disruption of intestinal barrier function, and cytotoxicity leading to cell death, which all result in impaired pig performance. Feed additives, such as mycotoxin-detoxifying agents, that are currently available often combine organic and inorganic sources to enhance their adsorbability, immune stimulation, or ability to render mycotoxins less toxic. In summary, mycotoxins present challenges to pig production globally because of their increasing occurrences in recent years and their toxic effects impairing the health and growth of pigs. Effective mycotoxin-detoxifying agents must be used to boost pig health and performance and to improve the sustainable use of crops.

## 1. Introduction

Mycotoxins are secondary metabolites that are naturally produced by fungi and may have toxic effects. For instance, mycotoxins may present negative effects when fed to livestock animals in contaminated feedstuffs. Mycotoxin contamination in feedstuffs can occur in farms, postharvesting, or during storage [1]. *Alternaria*, *Aspergillus*, *Cladosporium*, *Fusarium*, and *Penicillium* are among the most frequent genera of fungi to cause intoxications [1,2]. Studies have shown that feedstuffs and finished feeds are found to be contaminated with mycotoxins frequently and ubiquitously. More than 70% of feedstuffs and animal feeds produced worldwide are contaminated with at least one mycotoxin, where the most prevalent mycotoxins are deoxynivalenol (DON), aflatoxin B1, and fumonisins [1,3,4].

In pig production, mycotoxins are known to impair the health and growth of animals. Due to the toxic effects of mycotoxins, the Food and Drug Administration sets levels for mycotoxins in the United States. For nursery pigs, the aflatoxin concentration must not surpass 0.02 mg/kg, DON concentrations are advised not to surpass 1 mg/kg, and fumonisins must not surpass 10 mg/kg in the finished feeds [5,6]. Similarly, the European Commission stipulated 0.01 mg/kg as the upper limit for aflatoxin B1, 0.9 mg/kg as the advised upper limit for deoxynivalenol, and 5 mg/kg as the upper limit for fumonisins [7,8]. The economic losses derived from poor animal performance caused by mycotoxins are not the only financial impact. The economic losses of the three most frequent mycotoxins (aflatoxins, deoxynivalenol, and fumonisins) considering agriculture, livestock, and mitigation strategies (without considering the direct impact on human health) was estimated at $1.4 billion annually in the United States [9]. These concerning mycotoxin impacts on animal performance and economic losses are expected to be further aggravated by climate changes, with a higher prevalence or levels of mycotoxin contamination expected [10,11,12]. Therefore, the number of investigations into mycotoxin-detoxifying agents as feed additives that mitigate the toxic effects of mycotoxins is increasing. 

The use of mycotoxin-detoxifying agents as feed additives is advantageous for reducing the toxic effects of mycotoxins in pigs and, at the same time, may reduce the waste of crops and enable more sustainable use of feedstuffs. There are many mechanisms by which mycotoxin-detoxifying agents mitigate the toxic effects of mycotoxins in feeds. One such mechanism is by adsorption, where the mycotoxin interacts with another molecule (adsorbent) becoming not absorbable to the animal body. In the adsorbed form, the mycotoxin will be excreted in the feces and its toxic effects will be minimized in the animal. Another mechanism is to use these agents to boost immune function and gut health, making the animal less susceptible to the toxic effects of mycotoxins. These agents frequently include the use of prebiotics, probiotics, postbiotics, phytobiotics, and synbiotics [13]. 

For this review, aflatoxins, deoxynivalenol, and fumonisins were selected to be covered based on the impact of such mycotoxins in pig production (mycotoxin frequency, their toxic effects in pigs, and the existence of official regulations). In addition, this review covered mycotoxin-detoxifying agents, which are expected to be employed more frequently because of the growing mycotoxin contamination and the need to optimize the utilization of food and feed products.

## 2. Mycotoxin Occurrence

The initial mycotoxin contamination of feedstuffs and feeds can occur at the crop farm (before or during harvesting) or during storage, transportation, feed manufacturing, and even at the animal farm prior to consumption by pigs [1]. Mycotoxin contamination may also be influenced by the type of feedstuff, thus affecting the final contamination in pig feed. Therefore, it is important to measure the incidence and the concentration of mycotoxins in feedstuffs and feeds before pigs access them. Besides the initial contamination, other factors may allow for fungal development and, eventually, increase mycotoxin contamination. For instance, the occurrence of mycotoxins may differ depending on the geographic location, but this variation is likely to be influenced by the weather conditions. Global mycotoxin occurrence and concentration in feedstuffs and feeds used for pigs, as well as the factors influencing mycotoxin occurrence, testing, and results, are discussed in this section.

Mycotoxin contamination in feedstuffs and feeds is observed globally. Over ten years, it was observed that 88% of samples were contaminated with at least one mycotoxin in an investigation involving 100 countries [4]. A similar percentage was observed in an eight-year study comprising 82 countries, where 72% of samples were contaminated with mycotoxins [3]. Specifically, in pig feeds, 96% of samples were found to be contaminated with at least one mycotoxin globally [14]. Recent publications [3,4,14,15,16,17] regarding the occurrence of mycotoxins are summarized in Table 1.

Overall, the *Fusarium* toxins were the most frequently detected. Either fumonisins, deoxynivalenol, or zearalenone ranked first as the most detected mycotoxins across the studies [3,4]. The high frequency of *Fusarium* toxins was similarly observed when geographic regions were considered. Even in studies with a broad survey, including the assessment of emerging mycotoxins, which are frequently overlooked, zearalenone ranked in first place in terms of occurrence [14]; the exceptions were in sub-Saharan Africa and South Asia, where aflatoxin B1 ranked first [4]. 

Corn is a major feedstuff that is used globally for feeding pigs, making its investigation for mycotoxin contamination valuable when it comes to tracing and estimating the mycotoxin occurrence related to pig production. Among corn samples, the most frequent mycotoxins were fumonisins, followed by deoxynivalenol and zearalenone [3]. Alarmingly, according to Streit et al. [3], corn samples presented the highest occurrence (84%) and levels of mycotoxins across the samples tested, except for ochratoxin A. As expected, the same study found similar contamination levels between finished feed and corn, as corn is the main component in pig feed formulations. Comparable outcomes were observed by Gruber-Dorninger et al. [4], where the most frequently detected mycotoxins were fumonisins, deoxynivalenol, and zearalenone in both corn and finished feed. The same authors reported that finished feed samples showed a higher occurrence for most mycotoxins tested, as expected because of the combination of a variety of feedstuffs into the finished feed.

The effect of weather conditions on mycotoxin occurrence was observed in a trend of increased incidence across different mycotoxins in Southeast Asia, as severe rainy and dry seasons were observed in the same period [3]. Aflatoxin B1, deoxynivalenol, and fumonisins contamination were similarly related to weather conditions favoring crop contamination [4]. Due to confirming adverse weather as a contributing factor to mycotoxin occurrence, higher mycotoxin occurrence and contamination levels are expected in the future due to climate changes [18,19]. Due to climate changes, crops harvested out of the tropical area are expected to become more susceptible to fungal diseases and, thus, mycotoxin contamination [12]. Nevertheless, collected crop samples are not obligatorily harvested, stored, and processed in the same geographic location (country); thus, this can bring additional confounding factors to the obtained results if data are not attentively recorded.

Another factor that may influence the outcome observed in scientific studies is how data are presented and the number of mycotoxins tested. In a study assessing 320 fungal secondary metabolites, it was observed that the concentration of mycotoxins could be highly variable depending on whether the median or the average concentration was reported [17]. Such variation happened due to the occurrence of a few samples with exceptionally high concentrations pushing the average upward. Thus, reporting average concentrations may not truly represent the level of mycotoxin contamination in samples and the median may be more representative of a data set. Similar outcomes regarding average and median concentrations were also observed in a global survey of mycotoxin occurrence by Marin et al. [20]. To account for this variability, Gruber-Dorninger et al. [4] assessed the percentage of samples that surpassed the levels of mycotoxins set by the European Commission by considering 14 geographic regions. The percentage of samples that surpassed the recommended levels for all regions on average were 15.44, 2.29, 10.61, 10.42, and 0.79% for aflatoxin B1, fumonisins, zearalenone, deoxynivalenol, and ochratoxin A, respectively. These data show that at least 10% of samples were contaminated with aflatoxins, zearalenone, or deoxynivalenol at levels that may have detrimental effects on animal performance. However, the data presented by the authors did not allow for estimating co-contamination with mycotoxins. Thus, it is likely that the percentage of samples contaminated with any given mycotoxin exceeding the recommended levels by the European Commission and those with potentially detrimental effects will be higher than when considering only individual mycotoxins.

Furthermore, the number and types of mycotoxins tested may influence the outcomes. Mycotoxin testing on samples changes across studies and within the same study. For instance, Streit et al. [3] reported that the majority of wheat samples tested in eight years were tested for deoxynivalenol and zearalenone only. The lack of uniformity in sample testing may bias the outcomes of the studies. In addition, there are metabolites from fungal metabolism that are frequently overlooked in mycotoxin analyses. These metabolites are commonly known as “emerging” mycotoxins, which are currently unregulated and, thus, not considered in most of the tests [21]. The second class of commonly neglected mycotoxins is the “masked” or “modified” mycotoxins. The modified mycotoxins are those that underwent modification in their chemical structure and, thus, are not detected in conventional mycotoxin tests [22]. Even though most of the current reports regarding mycotoxin occurrence lack a screening for emerging and modified mycotoxins, current data are informative and may set guidelines for future investigations. 

In the following subsections, the occurrence of major mycotoxins (aflatoxins, deoxynivalenol, and fumonisins) are reviewed individually for a better understanding of their occurrence and their relationship with the stipulated levels in the United States and the European Union. Furthermore, the concomitant occurrence of mycotoxins is discussed in the last subsection because of its high incidence and increased likelihood of happening in the pig production scenario.

### 2.1. Aflatoxins

Aflatoxins are produced by fungi of the *Aspergillus* genus. *Aspergillus flavus* commonly contaminates grains and nuts with aflatoxins during the preharvest period [23]. *A. flavus* is known to produce aflatoxins B1 and B2. Another species, *Aspergillus parasiticus* can produce aflatoxins G1 and G2 in addition to the aflatoxins produced by *A. flavus* [23]. 

To limit the toxic effects of aflatoxins, the Food and Drug Administration sets action levels of 0.2, 0.1, and 0.02 mg/kg for pigs over 100 lb (about 45.5 kg), breeding animals, and immature animals (less than 4 months of age), respectively, for the sum of aflatoxins in the United States [6]. The European Commission has advisory limits for aflatoxin B1 contamination in feedstuffs and feeds for young pigs at 0.01 mg/kg and 0.02 mg/kg for older pigs [7]. The regulation of solely aflatoxin B1 is due to its greater toxicity, as well as its higher occurrence and concentration over other aflatoxins as a contaminant in feedstuffs and finished feeds. Therefore, only aflatoxin B1 is regulated in the European Union since it is indicative of contamination by other aflatoxins as well. Reinforcing such a regulation, in a study where most of the samples were European and from finished feeds, all samples which tested positive for aflatoxins were positive for aflatoxin B1 [17]. Additionally, aflatoxin B1 was detected as the most frequent mycotoxin among non-*Fusarium* toxins [3,4,15], showing the importance of setting guidance levels for such a frequent mycotoxin. Specifically for corn, contamination with aflatoxin B1 was correlated with increased temperatures and precipitation close to the silking and harvesting periods of corn [4].

### 2.2. Deoxynivalenol

Deoxynivalenol is a type B trichothecene, which is a naturally occurring metabolite of fungi from the *Fusarium* genus that may contaminate feedstuffs used in feed formulation. For instance, *Fusarium graminearum* and *Fusarium culmorum* are the main species that produce deoxynivalenol globally [24]. *Fusarium* toxins were the most frequent mycotoxins globally over the past ten years [4]. Deoxynivalenol was the most frequent mycotoxin, with more than two-thirds of samples of feedstuffs positive for it [4]. Among feedstuffs that were positive for deoxynivalenol, corn and wheat were the most frequently contaminated [4,15]. The contamination of crops with deoxynivalenol was correlated with mild temperatures and increased precipitation during the flowering and maturation periods [4]. Due to the high occurrence and deleterious effects of deoxynivalenol, there are governmental regulations in the United States and the European Union. The Food and Drug Administration has advisory levels recommending not surpassing 1 mg/kg of deoxynivalenol in feeds for pigs [5], whereas the European Commission stipulated 0.9 mg/kg of deoxynivalenol in feeds for pigs [8]. In European, Asian, and Pacific countries, *Fusarium* toxins are the most frequent, with the type B trichothecene, deoxynivalenol, ranking first [25]. A concerning outcome was observed in corn sampled over three years in the United States. Type B trichothecenes occurred in 78% of samples, with an average concentration of 1.2 mg/kg [15]. In finished pig feed, similar results were observed, where deoxynivalenol was detected in 88% of samples [14]. Although the deoxynivalenol concentration in contaminated samples can have a wide range (0–50 mg/kg), most samples are below 5 mg/kg [26]. However, 5 mg/kg is five-fold higher than the official guidelines in several countries. 

### 2.3. Fumonisins 

Fumonisins are also *Fusarium* toxins, being mainly found worldwide in crops contaminated with *Fusarium verticilliodes* and *Fusarium proliferatum* or locally by *Fusarium nygamai*, *Fusarium napiforme*, and *Fusarium globosum* [24]. The contamination of crops with fumonisins was correlated with increased temperatures and decreased rainfall during silking [4]. Fumonisins are mainly found as contaminants in corn and, as a consequence, in finished feeds [3]. The advisory levels set for fumonisins comprises the sum of fumonisins B1 and B2 at 5 mg/kg of finished feed for pigs in the European Union [8], and the sum of fumonisins B1, B2, and B3 at 10 mg/kg of finished feed in the United States [27]. Of interest, fumonisins ranked in order of occurrence are B1, B2, B3, and B4 [17], which justifies the use of the two (European Union) or the three (United States) most frequent mycotoxins among fumonisins as being indicative of their overall contamination.

### 2.4. Co-Occurrence

Even though most of the samples are under the limits and guidance levels set by the European and United States authorities, a considerable amount (38–64%) of samples are contaminated with more than a single mycotoxin [3,4]. This high frequency of co-contamination shows the need to investigate the association and the interaction of the effects of co-occurring mycotoxins in pigs. For instance, diets naturally contaminated with deoxynivalenol may impair pig growth at 0.6 mg/kg of diet, whereas for diets that are artificially contaminated with purified deoxynivalenol, growth impairment is observed at 1.8 mg/kg of diet [26].

Not surprisingly, and similar to the results observed for single mycotoxin occurrence, the mycotoxins most frequently found as co-contaminants for corn and finished feed were fumonisins, deoxynivalenol, and zearalenone for both global and regional assessments [4]. Among corn samples, 46% of the samples were co-contaminated [3]. A study that analyzed 524 samples of finished feeds for pigs found that 88% of the samples were contaminated with deoxynivalenol; in addition, 100% of deoxynivalenol-positive samples had a co-contaminating mycotoxin [14]. Out of the co-contaminants detected, nine mycotoxins were found in 90% or more of the samples along with deoxynivalenol (culmorin, 99%; zearalenone, 96%; brevianamide F, 95%; maculosin, 94%; enniatin B1, 92%; enniatin B, 91%; asperglaucide, 90%; emodin, 90%; moniliformin, 90%). Of note, eight of these co-contaminating mycotoxins are considered emerging mycotoxins. A concerning scenario was observed by Streit et al. [17], where all samples collected, mostly in Europe, were contaminated with mycotoxins. Yet more alarmingly, all samples had at least 7 and at most 69 co-contaminants detected.

Overall, mycotoxins are found to be contaminants in several feedstuffs, as well as in finished feeds, where they are detected ubiquitously. The majority of the samples that tested positive for mycotoxins were contaminated with multiple mycotoxins. With a few exceptions, *Fusarium* toxins were the most frequent mycotoxins detected, regardless of the sample types and geographic regions. Among all mycotoxins, deoxynivalenol, aflatoxins, and zearalenone were more frequently observed above the levels that may cause toxic effects in animals. Therefore, understanding the occurrence, as well as the toxic effects, of mycotoxins in pigs helps with finding the best choice of detoxification approach to be used in pig production.

## 3. Mycotoxin Toxicity

When ingested by pigs, mycotoxins can cause toxic effects that impair their health and growth. Even though zearalenone is among the most frequently detected mycotoxins, this review will further discuss aflatoxins, deoxynivalenol, and fumonisins because of the controversial effect of zearalenone in pig growth performance and the absence of any regulation for this mycotoxin in the United States, China, Brazil, and other key pig-producing countries.

### 3.1. Aflatoxins

Aflatoxins inhibit the RNA polymerase transcription of DNA to mRNA in the nucleus, reducing cell protein synthesis [28], and thus, increasing cell toxicity and death [29]. Aflatoxin B1 may suppress antigen-presenting cells by altering the function of dendritic cells and eventually reducing T-cell proliferation and differentiation [30]. Under chronic exposure, aflatoxin B1 can lead to immune suppression, hepatic damage, impaired growth, and may interact with the DNA, leading to neoplasia development [31,32]. Aflatoxin B1 shows higher toxicity and carcinogenic effects in comparison to other aflatoxins [23].

Indeed, aflatoxin B1 has caused detrimental effects on liver health and electrolyte balance in pigs [33]. Mycotoxins lead to impaired function and altered architecture of the liver and kidney [34,35]. The effects of mycotoxins in these two organs with important metabolic functions may influence cholesterol synthesis and, later, vitamin D activation, as well as the calcium and phosphorus balance [36,37]. Supporting the effect of mycotoxins on vitamin D metabolism, the toxic effects of aflatoxins on the kidney and vitamin D and calcium levels in poultry were previously demonstrated [38]. In the case of ingestion of aflatoxins, the liver has a central role in detoxification. The cytochrome P450 can either convert aflatoxins to its epoxide and more toxic form or to aflatoxins M1 and M2, which are less toxic [23]. Furthermore, aflatoxins cause impaired animal growth due to cytokine release [39].

### 3.2. Deoxynivalenol

The dietary intake of deoxynivalenol is known to reduce the feed intake and gain of pigs [40]. Pigs start showing reduced growth performance when fed at least 1 to 3 mg/kg of deoxynivalenol [20]. Specifically for naturally contaminated diets, concentrations of 1–2 mg/kg of deoxynivalenol reduce the feed intake and gain, where each additional 1 mg/kg of deoxynivalenol further reduces the gain by 8% in pigs [26]. 

Deoxynivalenol reduces feed intake in animals, especially in pigs, by modulating local serotonin and decreasing bowel movements [41,42], increasing satiety signaling [43], the release of proinflammatory cytokines [44], and potentially causing vomiting [45]. In mice, a deoxynivalenol-reduced feed intake was observed within 2 h after mycotoxin administration and with a dose-dependent response [43]. In addition to the reduced feed intake, growth is diminished by deoxynivalenol-induced disruption of the intestinal barrier and increased intestinal permeability via the activation of the mitogen-activated protein kinase pathway in pigs [46]. At the cellular level, deoxynivalenol has shown impairment on the translation of mRNA that may ultimately affect cell proliferation, development, and death [47,48,49,50], resulting in a reduction in feed intake and growth of pigs [40,51]. Furthermore, deoxynivalenol may impair the Wnt/β-catenin pathway, resulting in reduced mitosis in cells of the intestinal crypts [52]. Deoxynivalenol’s toxic effects on nutrient uptake include inhibiting SGLT-1 in the brush border membrane in the small intestine, which limits glucose absorption [53]. The decreased glucose uptake was demonstrated to be caused by a lower expression of the SGLT1 [54], as well as an inhibitor of the transporter [55]. At high concentrations, it was shown that deoxynivalenol at 10 mg/kg in feed can reduce the digestibility of essential amino acids in pigs [56]. As a result, the lower energy and nutrient intake and nutrient absorption, along with impaired cell metabolism, negatively impacted pig growth [57]. 

Besides the aforementioned toxic effects, deoxynivalenol can debilitate liver and kidney function [34,58]. Deoxynivalenol may also suppress the immune system at high doses or stimulate the immune system at low doses [47]. In pigs that are chronically fed deoxynivalenol-contaminated diets, an increased expression of interleukin-8 and glutathione peroxidase [51] and an increased serum total immunoglobulin A and specific immunoglobulin G [59] was observed. A summary of studies showing the toxic effects of deoxynivalenol on the growth performance of pigs is shown in Table 2.

### 3.3. Fumonisins

The main representative of the group composed of fumonisins is fumonisin B1. Fumonisin B1’s toxic effects are due to the inhibition of ceramide synthase, resulting in impaired sphingolipid metabolism with an accumulation of sphinganine [66]. The increase in sphinganine concentration in the liver and kidney is associated with induced cell apoptosis and mitosis, leading to fibrosis and nodular hyperplasia, respectively [67,68]. Therefore, the sphinganine-to-sphingosine ratio is frequently investigated as a biomarker for fumonisin B1 intoxication. 

In pigs, fumonisin B1 intoxication is associated with lung edema. The toxicosis can be observed within one week of feeding pigs a fumonisin-B1-contaminated diet, where respiratory distress and cyanosis signals are observed and may evolve to death [69]. Other effects of fumonisin B1 intoxication include cellular and humoral immunosuppression [70,71], hyporexia, and decreased weight gain [72]. In the gastrointestinal tract, fumonisin B1 disrupts the intestinal barrier [73] by affecting the tight junction function. Altogether, the impaired immune and barrier function make the intoxicated pig more susceptible to opportunistic pathogens [74].

### 3.4. Multiple Mycotoxin Toxicity

In the swine industry, multiple mycotoxins are detected, partially due to using a variety of feedstuffs in the finished feed and partially due to multiple fungi contamination. Therefore, pigs are more likely to face multiple mycotoxin toxicity in commercial farms than being challenged with a single mycotoxin. In general, animals are more sensitive to the toxic effect of mycotoxins when they are young and the pig is the domestic species showing the highest susceptibility to multiple mycotoxins, for instance, aflatoxins, deoxynivalenol, and fumonisins [75,76,77]. Overall, the toxic effect is stronger when mycotoxins are co-contaminants, even if the levels are below the governmental guidelines. 

In the review prepared by Alassane-Kpembi et al. [78], the interaction between mycotoxins was compared across in vitro toxicological studies. The concomitant challenges from aflatoxins (B1, B2, M1, and M2) showed a synergistic toxic effect, whereas aflatoxin B1 in combination with fumonisin B1 showed an antagonistic carcinogenic effect but a synergistic immunotoxic effect. For the interaction between aflatoxin B1 and trichothecenes, the effects were either synergistic or additive. In porcine kidney cells, aflatoxin B1 and deoxynivalenol showed synergistic cytotoxic damage to incubated cells [79]. Among trichothecenes, the interactive effects seem to be variable depending on the doses and proportions. In human intestinal cells, the combination of deoxynivalenol with its acetylated forms may result in synergistic or additive (3-acetyl-deoxynivalenol) to antagonistic (15-acetyl-deoxynivalenol) effects for low or high concentrations, respectively [80]. A similar study was conducted with intestinal porcine cells, where all trichothecene mixtures showed a higher inhibitory effect than the single mycotoxins [81]. Specifically for deoxynivalenol and 3-acetyl-deoxynivalenol, low, intermediate, and high doses presented antagonistic, additive, and synergic effects, respectively [81]. In intestinal porcine cells exposed to deoxynivalenol, fumonisin B1, and zearalenone, there was an additive cell toxicity, whereas deoxynivalenol and zearalenone had a synergic inhibitory effect on cell proliferation [78]. Ex vivo results in jejunal porcine explants showed a strong (2–14-fold increase) synergic effect of deoxynivalenol and nivalenol regarding inflammatory cytokine expression [82].

Alike in vitro and ex vivo outcomes, mixtures of aflatoxins, deoxynivalenol, and fumonisins have also shown different effects in animals, where mostly additive and synergistic effects were observed. In in vivo studies, aflatoxins and deoxynivalenol together cause liver damage and impair immune function, resulting in decreased growth in pigs [83,84]. Reinforcing the hypothesis of liver damage caused by mycotoxins, such mycotoxins reduced blood serum cholesterol in pigs fed a mycotoxin-contaminated diet [57]. A mycotoxin challenge with deoxynivalenol and aflatoxin B1 reduced the apparent ileal digestibility of nutrients in feeds in newly weaned pigs [57]. Additive or synergistic effects of deoxynivalenol and zearalenone were reported for parameters of immune function in mice and pigs [85].

In a meta-analysis prepared by Grenier and Oswald [86], publications were assessed for mycotoxin interactions. It was observed that aflatoxins and fumonisins mostly showed a synergistic effect in reducing the feed intake and weight gain in pigs. For aflatoxins in combination with deoxynivalenol, a synergistic effect in reducing cholesterol and glucose and in increasing white blood cells was observed, whereas there was an additive effect on creatine phosphokinase reduction but a less than additive effect on reducing weight gain. Lastly, the interaction between fumonisins and deoxynivalenol showed a synergistic effect on decreasing weight gain and increasing hepatic enzymes, but an additive effect for reducing feed intake. 

#### Estimation of Multiple Mycotoxin Toxicity

**Figure 1:**
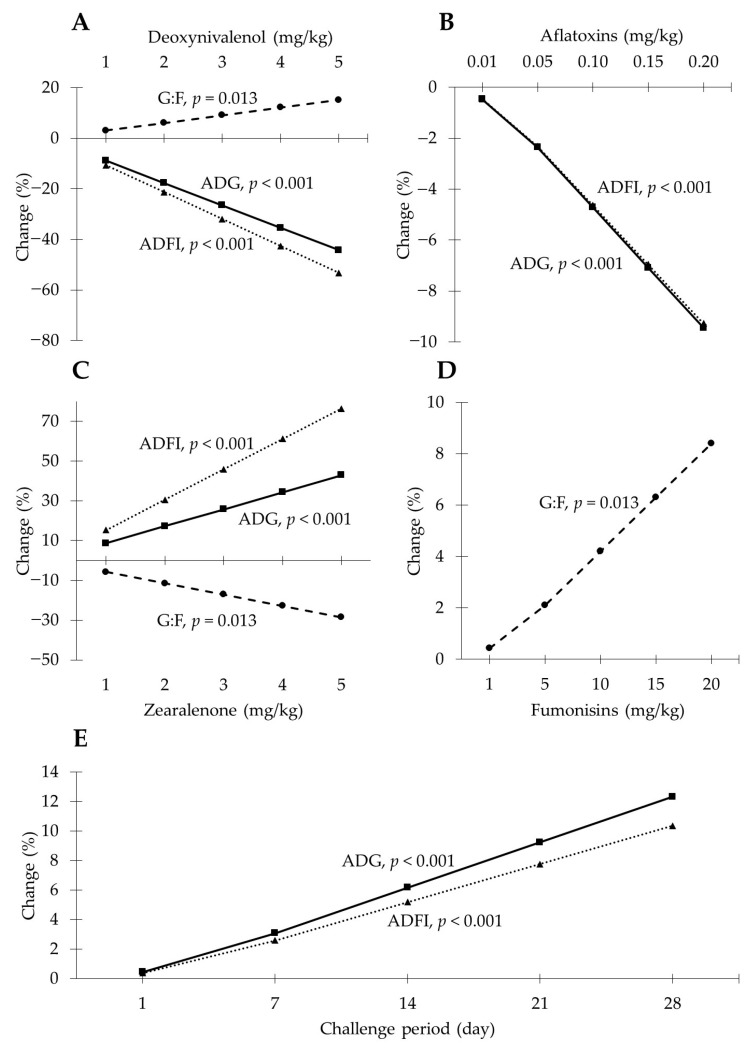
Parameter estimates of regression models for the percentual changes in growth performance variables in pigs challenged with multiple mycotoxins. (**A**) Percentual changes in the average daily gain (ADG), average daily feed intake (ADFI), and gain-to-feed ratio (G:F) caused by supplemental deoxynivalenol in the feed when all other variables remained constant. (**B**) Percentual changes in the ADFI and G:F caused by supplemental aflatoxins in the feed when all other variables remained constant. (**C**) Percentual changes in the ADG, ADFI, and G:F caused by supplemental zearalenone in the feed when all other variables remained constant. (**D**) Percentual changes in the G:F caused by supplemental fumonisins in the feed when all other variables remained constant. (**E**) Percentual changes in the ADG and ADFI caused by increasing the days of the challenge period when all other variables remained constant.

When using naturally contaminated feedstuffs in research trials, pigs are likely to be challenged with multiple mycotoxins, although one is of main interest and/or is above the advisory guidelines. Thus, this section aimed to estimate the individual contribution of mycotoxins in a multiple mycotoxin challenge. Based on the published mycotoxin studies performed by our research group, it was possible to estimate the parameters that would influence the percentual changes in growth performance variables in pigs under multiple mycotoxin toxicity due to supplemental levels of individual mycotoxins (Figure 1) [13,34,57,65,83,84,87,88]. The reason for choosing the studies performed by our research group is due to similarities in the pig genetics, environment (research facilities), and feedstuffs used (sometimes the same across studies). The candidate parameters included in the selection procedure for finding the best model were the supplemental mycotoxin concentrations (mg/kg) for deoxynivalenol, aflatoxins, zearalenone, and fumonisins, as well as the average initial body weight (kg) in the challenged and non-challenged pigs, phase (either nursery or grower), and duration of the challenge period in days. The supplemental mycotoxin concentrations used were the differential concentration among diets of pigs challenged or not with mycotoxins within each study. The selection of parameters was performed with the GLMSELECT procedure of SAS (version 9.3, Cary, NC, USA) using the STEPWISE statement. Then, the estimates for the selected parameters were obtained with the REG procedure. The estimations generated were based on supplemental mycotoxin concentrations ranging from 0 to 4.46 mg/kg for deoxynivalenol, from 0 to 0.22 mg/kg for aflatoxins, from 0 to 0.75 mg/kg for zearalenone, from 0 to 14 mg/kg for fumonisins, and from 21 to 48 days for the challenge period.

The results obtained showed that the increase in body weight (BW) of pigs during the study period was diminished (−8.8%) by a supplemental 1 mg/kg of DON in the feed and a supplemental 0.01 mg/kg of aflatoxins in the feed (AF; −0.4%), but it was increased by a supplemental 1 mg/kg of zearalenone in the feed (ZEA; +8.5%), and the duration in days of the challenge (day; +0.4%), whereas there was no influence from the supplemental fumonisins (FUM) in the feed, the initial body weight of the non-challenged pigs, the initial body weight of the challenged pigs, or the phase. The adjusted *R*^2^ for the equation generated (BW = −15.0 − 8.8 × DON − 0.4 × AF + 8.5 × ZEA + 0.4 × day) was 0.83 (*p* < 0.001).

The average daily gain (ADG) of pigs was diminished (−8.9%) by a supplemental 1 mg/kg of deoxynivalenol and a supplemental 0.01 mg/kg of aflatoxins (−0.5%), but it was increased by a supplemental 1 mg/kg of zearalenone (+8.6%) in the feed, and each day of challenge duration (+0.4%), whereas there was no influence from the supplemental fumonisins in the feed, the initial body weight of the non-challenged pigs, the initial body weight of the challenged pigs, or the phase. The adjusted *R*^2^ for the equation (ADG = −15.6 −8.9 × DON − 0.5 × AF + 8.6 × ZEA + 0.4 × day) generated was 0.83 (*p* < 0.001).

The average daily feed intake (ADFI) of pigs was diminished by a supplemental 1 mg/kg of deoxynivalenol (−10.7%) and a supplemental 0.01 mg/kg of aflatoxins (−0.5%) in the feed, but it was increased by a supplemental 1 mg/kg of zearalenone (+15.3%) in the feed and each day of challenge (+0.4%), whereas there was no influence from the supplemental fumonisins, the initial body weight of the non-challenged pigs, the initial body weight of the challenged pigs, or the phase. The adjusted *R*^2^ for the equation generated (ADFI = −9.1 −10.7 × DON − 0.5 × AF + 15.3 × ZEA + 0.4 × day) was 0.86 (*p* < 0.001).

The gain-to-feed ratio (G:F) of pigs was diminished by a supplemental 1 mg/kg of zearalenone (−5.7%) in the feed but it was increased by a supplemental 1 mg/kg of deoxynivalenol (+3.0%) and a supplemental 1 mg/kg of fumonisins (+0.4%) in the feed, whereas there was no influence from the supplemental aflatoxins, the initial body weight of the non-challenged pigs, the initial body weight of the challenged pigs, the phase, or the days of the challenge. The adjusted *R*^2^ for the equation generated (GF = −6.7 + 3 × DON + 4 × FUM − 5.7 × ZEA) was 0.48 (*p* = 0.013).

The observed decreases in body weight gain, average daily gain, and average feed intake caused by deoxynivalenol and aflatoxins were expected because of the toxic effects of these mycotoxins, as mentioned before: impaired cell metabolism, nutrient utilization, and performance in pigs. Deoxynivalenol and fumonisins increased the gain-to-feed ratio, increasing the efficiency of the conversion of nutrients into body tissues. The improved efficiency may happen as a result of the reduced body weight and feed intake in challenged pigs, which may become more efficient in using nutrients from feed [89]. Even though results regarding the increase in body weight gain, average daily gain, and average daily feed intake caused by zearalenone are controversial, similar outcomes were previously reported in studies with the purified toxin, along with a decrease in gain-to-feed ratio [90,91]. The lack of significant effect of fumonisins on body weight gain, average daily gain, and average daily feed intake could be due to the average concentration of fumonisins across studies, which was 2 mg/kg. This average concentration is below the established guidelines in Europe and the United States of 5 and 10 mg/kg of fumonisins, respectively [8,27]. Thus, it may explain the absence of detrimental effects in pigs in the current model. Unexpectedly, it was observed that an increase in the number of days of mycotoxin challenges actually increased body weight gain, average daily gain, and average daily feed intake. This result could be because of the challenge period included in the model (from 21 to 48 days), when pigs are facing the chronic effects of mycotoxins. Therefore, an increase in days chellenged could reduce the toxic effects of mycotoxins as pigs may get acclimated to the mycotoxins.

Altogether, the elevated prevalence of major mycotoxins and their toxic effects, as well as the stipulated levels by governmental institutions, make investigations assessing the efficiency of feed additives that can be used as mycotoxin-detoxifying agents for pigs quite valuable.

## 4. Mycotoxin-Detoxifying Agents

To enable the use of contaminated feedstuffs and feeds for animal consumption by diminishing or avoiding the toxic effects of mycotoxins, mycotoxin-detoxifying agents can be employed as feed additives. There are many mycotoxin-detoxifying agents with different mechanisms of action. For example, some agents are used to mitigate the toxic effects of mycotoxins in pigs via adsorption, enhancing immune functions, and as detoxifiers (such as microorganisms) [92,93]. Particularly for deoxynivalenol, it is still a challenge to find an efficient mycotoxin-detoxifying agent that can counteract its toxic effects. In Table 3, the effects of mycotoxin-detoxifying agents on the growth performance of pigs challenged with deoxynivalenol, alone or in combination with other mycotoxins, are summarized.

### 4.1. Inorganic Compounds

Activated charcoal has been known for a long time as a potent adsorbent for multiple mycotoxins [94,95]. However, because of its nonspecific binding (inclusive of nutrients in feed), activated charcoal should have its use restrained to cases of acute intoxication with high concentrations of mycotoxins where there is an imminent risk of severe toxicosis or death [96]. 

Aluminosilicates have a comparatively lower adsorbability to mycotoxins, but it is considerably enhanced for the hydrated sodium calcium form, particularly for aflatoxins [97]. The adsorbability of aluminosilicates is also enhanced in the case of the association with organic compounds [98]. The adsorbability of several inorganic adsorbents to aflatoxin B1 was tested in an in vitro model, where a carbon-and-aluminosilicate-based product, phyllosilicates (Attapulgite, Greek bentonite, sodium bentonite, activated bentonite, Indian bentonite, Myco-AD AZ), and tectosilicates (Clinoptilolite, CAB 70) were tested [98]. At 1 and 10 µg/mL of aflatoxin B1, the carbon-and-aluminosilicate-based product and phyllosilicates showed a binding efficiency of approximately 80% or more to aflatoxin B1, whereas a lower efficiency (61 to 8%) was observed for tectosilicates. In vivo, bentonites and hydrated sodium calcium aluminum silicates could effectively overcome the toxic effects of aflatoxins in pigs [99]. However, diet formulation may need adjustments as sodium calcium aluminum silicates may also interact with minerals in feed [33].

Bentonites show low adsorbability to deoxynivalenol (3.2%) in vitro in comparison to other mycotoxins, such as aflatoxins (92.5%) [100]. The higher polarity of aflatoxins when compared to deoxynivalenol is likely the cause for the reduced binding ability of bentonites to deoxynivalenol [97,101]. Diatomaceous earth has an intermediate adsorbability to mycotoxins, though it is amid the inorganic materials with the highest adsorbability to *Fusarium* mycotoxins [97].

A carbon-and-aluminosilicate-based product, the same as aforementioned, was tested in a different study for fumonisin B1 adsorption, showing higher adsorbability among all mycotoxins tested under different pHs (100%) [102]. Fumonisin B1 adsorption by other aluminosilicates (bentonite and zeolite) and diatomaceous earth (celite) showed higher efficiencies in an acidic environment up to 100% (bentonite) but was greatly decreased to 26% in a neutral environment (bentonite) [102].

### 4.2. Yeast

The use of yeast in the livestock feed industry emerged from the abundance of by-products from the food industry. One such use of yeast as a feed additive is as a mycotoxin adsorbent. Cellular components from yeasts, namely, the cell wall and intracellular content, may be used as feed additives. The cell wall is a complex structure of carbohydrates composed of glucans, mannans, and chitin [103,104]. Out of the carbohydrates composing the yeast cell wall, glucans were identified as a fundamental element in the interaction and adsorption of mycotoxins [65,105,106], as well as demonstrating prebiotic properties [107,108]. Furthermore, the α- and β-D-glucans are elements of the yeast cell wall that may selectively interact with enterocytes and microbes, modulating the pig immune function and microbiome, respectively [109]. Yeast cell wall interactions with enterocytes and microbes have further effects, resulting in diminished oxidative stress [110,111]. Therefore, a dietary yeast cell wall can have advantageous prebiotic properties by facilitating the metabolism and growth of beneficial microorganisms, resulting in an improved intestinal barrier, health, and immunity [112,113]. Furthermore, the inclusion of yeast culture, as a probiotic, may increase the carbohydrate fermentation in the intestinal lumen, providing beneficial metabolic products, such as peptides and organic acids, and improving the nutrition and health of pigs [108]. The feed additives containing fermentation extracts derived from yeast metabolism may have similar beneficial effects to yeast metabolic products [13]. Nevertheless, the fermentation extracts will be present in a limited amount in the additive instead of being produced in the intestinal lumen. The use of inactivated yeast may enable yeast cell wall interaction with enterocytes and with mycotoxins, similar to using yeast culture or yeast cell wall extract, improving intestinal health and reducing mycotoxin toxicity. An enhanced intestinal health and immune response are seen after the inclusion of a feed additive with *Saccharomyces cerevisiae* as either a yeast culture or inactivated yeast [13,57] through a reduction in CD4+ activation and eventual IFN-γ production [108,114], thus, reducing inflammation and enhancing enterocyte integrity [115]. Feed additives with yeast culture have shown enhanced animal health, gut integrity, and digestibility of nutrients in feed, along with decreased *Escherichia coli* shedding in feces, improving the performance of non-challenged pigs [108,114].

The yeast cell wall, and more particularly β-D-glucans, have robust adsorbability to aflatoxin B1 and zearalenone but with restricted efficiency to deoxynivalenol [100,106]. The detoxification of aflatoxin B1 by *Saccharomyces cerevisiae* strains was, on average, 65% after 24 h of incubation [116]. 

Yeast- and algae-derived β-glucans can show higher adsorbing abilities than mineral adsorbents, especially under alkaline pH for deoxynivalenol and zearalenone [101,106,117]. However, this limited (but existing) adsorbability of yeast cell wall components, such as β-D-glucans and glucomannans, to *Fusarium* toxins [118,119] can be an advantage in comparison to inorganic binders. Indeed, in an in vitro study simulating the gastrointestinal tract of pigs, yeast cells could adsorb 23% of deoxynivalenol, whereas bentonite, cellulose, and activated charcoal could adsorb 3, 12, and 14% of deoxynivalenol, respectively [100]. In another study, the detoxification of deoxynivalenol by *Saccharomyces cerevisiae* strains was, on average, 33% after 24 h of incubation [116]. While low, the binding ability of the yeast cell wall seems to be the highest with deoxynivalenol. In addition, processing yeast into yeast cell walls may result in improved adsorbability to mycotoxins [97]. There are few investigations on the yeast cell wall as a sole mycotoxin-detoxifying agent to mitigate the toxic effects of deoxynivalenol in pigs. Yeast cell wall’s minor effects in ameliorating health and growth in deoxynivalenol challenged pigs are likely the reason for the small number of studies [40,120], as the yeast cell wall plays an accessory effect as a deoxynivalenol-detoxifying agent. Specifically related to the deoxynivalenol challenge, the yeast cell wall seems to have lower immune-modulatory effects than the whole cell in newly weaned pigs, indicating that yeast fermentation products may have a major function in mitigating the toxic effects of deoxynivalenol in the gut in comparison to the yeast cell wall [13]. Such an outcome could be due to the reduced immune response and improved maintenance of gut integrity, both with a major role in pigs’ susceptibility to deoxynivalenol, instead of the adsorbability of yeast fermentation products [13].

In contrast to deoxynivalenol, the detoxification of fumonisins (another *Fusarium* toxin) by *Saccharomyces cerevisiae* strains was, on average, 72% after 24 h of incubation [116]. However, the detoxifying capacity of yeast is not high for all species and strains. Naturally occurring bacteria and yeast in silage were tested for their fumonisin-detoxifying capacity, where bacteria showed up to a five-fold higher detoxification in comparison to yeast [121]. In most studies including fumonisins and yeast-based products, other mycotoxins are co-contaminants [57,65,87]. The lack of studies was probably due to the high efficiency of mycotoxin-detoxifying agents with other components, as discussed above in the case of inorganic adsorbents. Only one in vivo study assessing yeast mitigation effects in pigs challenged by fumonisin as a single toxin was found in our survey of scientific publications. A recent study was found where pigs were challenged with fumonisins and three different products, with one being a yeast-based product, where it showed a recovery in the growth performance and the sphinganine-to-sphingosine ratio [122]. Nevertheless, the other two products tested were not specific detoxifying agents for fumonisins.

### 4.3. Bacteria

Similarly, the investigation of alternative uses of by-products rich in bacteria has emerged from the dairy and baking industries. The binding ability of *Lactobacillus casei* to aflatoxin B1 was shown to depend on the cell wall structure, where the live cell or cell wall fractions had similar adsorbability but heat treatment decreased its adsorbability [123]. In this case, the damage to the protein structure, which aflatoxin B1 has a high affinity to, by heat treatment was claimed as the reason for *L. casei* losing its adsorbability. The adsorption of aflatoxin B1 by live cells of *L. casei* (10^9^ CFU/day) caused conformational changes in the bacterial cell wall, reduced the intestinal absorption of aflatoxin B1, and overcame the detrimental effects observed in mice [123]. The detoxification of aflatoxin B1 by *Lactobacillus* species was, on average, 60% after 24 h of incubation [116].

Feeding deoxynivalenol to weanling pigs may modulate the gastrointestinal microbiome [124], indicating that the gastrointestinal microbiome can change to avoid deoxynivalenol toxicity. Among the microbial genera with deoxynivalenol-detoxifying capacity are Eubacteria, *Anaerofilum*, *Collinsella*, *Bacillus*, and Clostridiales [125]. Least commonly but also of interest, aerobic Gram-positive bacteria may catalyze the de-epoxidation reaction as described for *Nocardioides* and even aerobic Gram-negative bacteria, such as *Devosia*, which are generally characterized as casual degraders [126]. The detoxification of deoxynivalenol by *Lactobacillus* species was, on average, 30% after 24 h of incubation [116]. However, it was observed that mycotoxin-detoxifying agents with Gram-positive bacteria can adsorb deoxynivalenol rather than converting it to its less toxic compounds [127]. Gram-positive bacteria, such as *Streptococcus* and *Enterococcus*, have shown adsorbability up to 33% toward deoxynivalenol in corn silage [127], whereas *Lactobacillus helveticus* could adsorb 55%, and heat-inactivated *Lactobacillus plantarum* could adsorb up to 71% of deoxynivalenol in liquid media [128]. Following up on this study, the adsorbability of deoxynivalenol by several Gram-positive bacteria was tested and an overall increase in the adsorbability was observed after heat treatment [128]. Such lack of species-specific adsorption across Gram-positive bacteria strains, along with the increased adsorption after heat-inactivation of bacteria, suggest that the bacterial cell wall may be involved in the mycotoxin–bacteria interaction. The role of the cell wall of Gram-positive bacteria in the adsorption of deoxynivalenol was later proved by Zou et al. [129], where similar adsorption by *L. plantarum* was demonstrated by either the cell pellet or cell wall, but no adsorption was observed by the cell extract or its fermentation product. Of interest, the deoxynivalenol–*L. plantarum* interaction could be preserved when incubated in simulated gastric and intestinal fluids for 30 min to 4 h [129]. The detailed mechanism by which deoxynivalenol adsorption by the cell wall of Gram-positive bacteria happens is not fully elucidated. However, it can be inferred that the increase in temperature causes protein denaturation, leading to pore formation, which could enhance the surface area with a binding ability [130]. Moreover, the hydrophobicity of the cell wall from *Lactobacillus* [130] is enhanced by heat treatment [131], which may facilitate deoxynivalenol adsorption [129]. Ex vivo, the culture supernatant from *L. plantarum* after heat treatment could improve the architectural damage to intestinal villi caused by deoxynivalenol in jejunal explants of pigs [132]. Similarly, jejunal explants of pigs treated with *Lactobacillus rhamnosus* (10^9^ CFU/mL) before deoxynivalenol exposure showed a reduction in paracellular permeability, the production of proinflammatory cytokines (tumor necrosis factor alpha and interleukin-8), and the activation of mitogen-activated protein kinases [133]. However, due to deoxynivalenol’s small chemical structure and low polarity, finding compounds with strong adsorbability to deoxynivalenol and with the potential to mitigate its toxic effects is a current challenge [63,64,101]. 

Following the same line, the mechanism of interaction between fumonisins and the cell wall of Gram-positive bacteria was investigated. The adsorbability of fumonisins by the cell wall of Gram-positive bacteria increases with a further breakdown of the cell wall structure if the peptidoglycan remains intact [134]. The peptidoglycan was recognized as the component from the bacterial cell wall, which adsorbs the tricarballylic acid chain from fumonisins [134]. The detoxification of fumonisins by *Lactobacillus* species was about 70% after 24 h of incubation [116]. However, no in vivo studies were found that tested bacterial mitigating properties in pigs challenged with fumonisins as a single mycotoxin. It is likely that the absence of studies with bacterium-based products in pigs challenged with fumonisins, as seen for yeast-based products, is because of the high efficiency of inorganic adsorbents.

### 4.4. Others

Phytobiotics, as plant extracts, and antioxidants are often included in mycotoxin-detoxifying agents’ formulas to diminish the oxidative stress caused by mycotoxins and enhance intestinal health. Algae derivatives may present antioxidant properties under mycotoxin challenge, resulting in an enhanced gain in poultry [135]. In pigs, algae extracts improved nutrient and energy digestibility, decreased *E. coli* counts in feces, and improved growth performance [136]. Feed additives containing calcium propionate may reduce intestinal pH, increase the digestibility of nutrients in feed, and improve intestinal health [137]. Furthermore, calcium propionate is an organic acid with the ability to impair fungal colonization or growth in feeds [75]. Considering animals under mycotoxin challenge, calcium propionate improved liver health, reduced mycotoxin concentration in organs, and recovered the growth performance in broilers [138]. In pigs, a mycotoxin-detoxifying agent combining hydrated sodium calcium aluminum silicate, calcium propionate, and calcium formate was tested to mitigate the toxic effects of multiple mycotoxins (zearalenone, aflatoxin, and ochratoxin) [139]. As a result, this mycotoxin-detoxifying agent promoted intestinal health, nutrient digestibility and absorption, and gain [139]. Plant derivatives can be used to reduce the detrimental effects caused by mycotoxins in pigs more specifically in the gastrointestinal tract. One example is milk thistle, which can reduce inflammatory signaling in vitro by reducing tumor necrosis factor alpha, resulting in reduced cell death [140]. Another example is rosemary, which may neutralize and destroy *Fusarium* fungi [141]. 

Overall, the use of mycotoxin-detoxifying agents containing multiple components to mitigate the toxic effects of mycotoxins has shown more benefits in comparison to those with single components, particularly in the case of deoxynivalenol challenge [115]. 

## 5. Conclusions

Mycotoxins have a high prevalence in feedstuffs and swine feeds globally, which may impair the health and growth of pigs. It is important to consider the increased negative effects of mycotoxins when they are co-contaminants, including the occurrence of emerging and masked mycotoxins. Studies with purified mycotoxins may show lower toxicity in comparison to mycotoxins that naturally contaminated feeds. Therefore, future studies must have a broad mycotoxin screening that allows for the correct interpretation of the data and the projection of trends. Mycotoxin-detoxifying agents include adsorbents, health stimulants, and detoxifiers. The frequent co-contamination with mycotoxins in feedstuffs and feeds intended for pig consumption makes mycotoxin-detoxifying agents targeting multiple mycotoxins preferable.

## Figures and Tables

**Table 1:** Frequency and occurrence of single or multiple mycotoxins according to sample type and origin.

Period of Sampling	Samples	Origin	Top Four Mycotoxins Detected (Frequency)	Samples Positive to		Reference
				Single Mycotoxin	Multiple Mycotoxins	
2004 to 2011	17,316—feed and feedstuff	Global	DON (55%), FUM (54%), ZEA (36%), AFL (27%)	72%	38%	[3]
2008 to 2019	74,821—feed and feedstuff	Global	DON (64%), FUM (60%), ZEA (45%), AFB1 (23%)	88%	64%	[4]
2016	595	United States	Type B trichothecenes (85%), FUM (61%), ZEA (51%), AFL (5%)	-	≥85%	[15]
2017	733	United States	Type B trichothecenes (78%), FUM (43%), ZEA (32%), AFL (1%)	-	≥78%	[15]
2018	147—corn and corn derivatives	United States	Type B trichothecenes (56%), FUM (64%), ZEA (31%), AFL (10%)	-	≥56%	[15]
2011 to 2014	1384—corn, corn silage, cereals, feed	Poland	DON (95%), NIV (85%), T2 (79%), HT2 (85%)	68%	-	[16]
2014 to 2018	524—finished feed for pigs	Global	ZEA (96%), brevianamide F (95%), culmorin (94%), maculosin (94%)	≥96%	88%	[14]
2010 to 2012	83—feed and feedstuff	Europe, America, Australia	Beauvericin (98%), ennitatins (96%), DON (89%), emodin (89%)	100%	100%	[17]

DON, deoxynivalenol; FUM, fumonisins; ZEA, zearalenone; AFL, aflatoxins; AFB1, aflatoxin B1; NIV, nivalenol; T2, T2 toxin; HT2, hydroxy-T2 toxin. The dash “-” is used when the information could not be retrieved from the publication.

**Table 2:** Toxic effects of deoxynivalenol challenges, alone or in combination with other mycotoxins, on the growth performance of nursery pigs.

Mycotoxin, Concentration (mg/kg)	*n*	BW Range (kg)	Duration (days)	Change in Growth Performance (%)			Reference
				ADG	ADFI	G:F	
DON, 1 (purified)	120	10–20	23	−0.4	−1.1	−1.9	[60]
DON, 2.3	36	7.5–16.5	21	−18.4	−15.9	−4.2	[57]
DON, 2.6 (purified)	120	7–10	14	−12.2	−11.3	0.0	[60]
DON, 3.2	60	8.2–20.6	34	−11.7	−5.9	−5.6	[13]
DON, 3.5 (purified)	16	8–?	35	−19.2	−19.8	+0.8	[61]
DON, 3.55	24	6–11	21	−17.8	−14.6	−3.8	[62]
DON, 4.2	126	13.4–22.4	21	−18.9	−12.0	−7.8	[63]
DON, 4.61	20	6.9–11.0	14	−41.0	−21.5	−23.7	[64]
DON, 7.38 (purified)	10	19.3–40.1	28	−30.2	−7.1	−24.8	[35]
AFL, 0.18; FUM, 9; DON, 1	48	6–29	36	−15.8	−18.5	+2.9	[65]
DON, 4.45; FB1, 0.76; ZEA, 0.44	780	22.8–103.8	115	−12.0	−8.7	−2.4	[40]

BW, body weight; ADG, average daily gain; ADFI, average daily feed intake; G:F, gain-to-feed ratio; AFL, aflatoxins; DON, deoxynivalenol; FUM, fumonisins; ZEA, zearalenone; FB1, fumonisin B1.

**Table 3:** Effects of mycotoxin-detoxifying agents on the growth performance of pigs challenged with deoxynivalenol, alone or in combination with other mycotoxins.

Mycotoxin, Concentration (mg/kg)	*n*	BW Range (kg)	Duration (days)	Mycotoxin-Detoxifying Agent			Change in Growth Performance (%)			Reference
				Inorganic	Yeast	Other	ADG	ADFI	G:F	
DON, 1	120	10 to 20	23	Acid-activated bentonite and clinoptilolite	Yeast cell wall	Organic acids	+5.0	+4.7	0.0	[60]
DON, 2.3	36	7.5 to 16.5	21	-	Hydrolyzed yeast cell wall	Organic acids, vitamins, and essential oils	+5.4	+5.6	+9.0	[57]
DON, 2.6	120	7 to 10	14	Acid-activated bentonite and clinoptilolite	Yeast cell wall	Organic acids	+28.9	+19.8	+7.5	[60]
DON, 3.2	36	8.19 to 20.73	34	Bentonite	Yeast culture	Diatomaceous earth and kelp	+11.8	+4.7	+6.0	[13]
DON, 3.2	36	8.19 to 20.55	34	Organo-aluminosilicate clays	Yeast cell walls	Plant extracts, triglycerides, calcium propionate, and antioxidants	+7.4	+3.1	+4.5	[13]
DON, 3.2	36	8.21 to 20.44	34	Sepiolite and bentonite	Inactivated yeast and fermentation extracts	Propyl gallate, calcium propionate, milk thistle seed, rosemary, licorice root, and boldo	+4.1	−0.8	+3.0	[13]
DON, 3.82	30	6.9 to 11.2	14	-	-	Sodium metabisulfite, organic acids, vitamins, and amino acids	+60.0	+13.8	+36.2	[64]
DON, 4.2	126	13.4 to 22.4	21	Adsorbent clays	-	Preservatives	−10.1	−9.0	−1.6	[63]
DON, 4.41	30	7.0 to 10.9	14	-	Yeast extract	Live bacteria, enzymes, and plant extracts	+19.5	+12.8	+5.2	[64]
DON, 4.66	30	6.9 to 10.6	14	-	Yeast glucomannan	-	+1.8	+6.9	−8.6	[64]
DON, 4.65	30	6.9 to 10.7	14	Aluminosilicate	-	-	+7.3	0.0	+6.9	[64]
DON, 4.45; FB1, 0.76; ZEA, 0.44	780	22.9 to 104.6	115	-	-	Sodium metabisulfite, organic acids, vitamins, and amino acids	+9.1	+5.6	+3.0	[40]
DON, 4.45; FB1, 0.76; ZEA, 0.44	780	22.8 to 103.3	115	Hydrated sodium calcium aluminosilicate and silicon dioxide	Hydrolyzed yeast	-	+4.5	+3.4	+0.8	[40]
AFL, 0.18; FUM, 9; DON, 1	48	6 to 29	36	Hydrated sodium calcium aluminosilicate	Yeast cell wall	Algae	0.0	−3.9	+4.3	[65]

BW, body weight; ADG, average daily gain; ADFI, average daily feed intake; G:F, gain-to-feed ratio; AFL, aflatoxins; DON, deoxynivalenol; FUM, fumonisins; ZEA, zearalenone; FB1, fumonisin B1.

## Data Availability

This is a review paper and thus the data presented in this study are openly available in published papers listed in References.
